# Hedione Reduces Subjective Vicarious Stress

**DOI:** 10.3389/fnbeh.2019.00297

**Published:** 2020-01-17

**Authors:** Anika Pützer, Martin Brüne, Hanns Hatt, Oliver T. Wolf

**Affiliations:** ^1^Department of Cognitive Psychology, Faculty of Psychology, Institute of Cognitive Neuroscience, Ruhr University Bochum, Bochum, Germany; ^2^International Graduate School of Neuroscience, Ruhr University Bochum, Bochum, Germany; ^3^LWL University Hospital Bochum, Department of Psychiatry, Division of Social Neuropsychiatry and Evolutionary Medicine, Ruhr University Bochum, Bochum, Germany; ^4^Cell Physiology, Faculty of Biology and Biotechnology, Ruhr University Bochum, Bochum, Germany

**Keywords:** stress, empathic stress, stress contagion, vicarious stress, hedione, chemosensory communication, chemosignal

## Abstract

Observing another person in a stressful situation can cause a full-blown physiological stress response in the observer, which is referred to as empathic stress. One way through which stress-related information might be transmitted between individuals under conditions of empathic stress is chemosensory communication. In the present study, we investigated whether the odorant Hedione, as a potential chemosignal, affects the empathic stress response at a physiological and psychological level. For this purpose, two experiments were designed, each testing one group of participants in an odor-free room and a second group in a room scented with Hedione. In Experiment 1, 60 participants (25 males) watched a video of an unknown female participant in the Trier Social Stress Test (TSST). In Experiment 2, 37 free-cycling females watched a live video of a male participant in the TSST. Observers’ psychological and physiological stress response was captured *via* repeated measurements of salivary cortisol, alpha-amylase, and self-report ratings. Empathy with the stressed participants was assessed on the dimensions of personal distress and empathic concern of the Emotional Response Scale (ERS). Our results show no substantial physiological stress response in the observers and no effect of Hedione on physiological stress measures. Further, in Experiment 1, there was no subjective stress elicited by the video and no effect of Hedione. In Experiment 2, the observation was perceived as stressful and Hedione reduced subjective vicarious stress. The subjective stress response was associated with the Observers’ direct personal distress, but not with their empathic concern for the target in both experiments. Based on the findings presented above, we conclude that under conditions of empathic stress, Hedione alleviates subjectively perceived stress felt when observing another person being stressed, while leaving empathic concern for the target unaffected. In this regard, future research is warranted to clarify the underlying mechanisms of this effect.

## Introduction

Stressful events trigger a cascade of physiological and psychological processes to optimally deal with the situation. The direct encounter of a stressful event is, however, not a prerequisite for an acute stress response to appear. At least partly, a stress response can arise from the mere observation of another person (target) in a stressful situation. This phenomenon is referred to as stress contagion (Waters et al., 2014, 2017; Dimitroff et al., 2017) or empathic stress (Engert et al., 2014). Engert et al. (2014) differentiate two components of empathic stress. Vicarious stress describes the observer’s stress response being irrespective of the target’s response. Stress resonance (Engert et al., 2019) indicates an association between the stress responses of observer and target. Both components were found to be positively associated with the observer’s tendency to empathize with the target, to be modulated by the emotional closeness of target and observer and to be unaffected by observer sex (Engert et al., 2014).

Empathic stress manifests at different levels of the physiological stress response. Stress resonance of the sympathetic nervous system (SNS) was, for instance, repeatedly reported for mother-infant dyads (Ebisch et al., 2012; Manini et al., 2013; Waters et al., 2014, 2017). Furthermore, people observing a target being stressed in the laboratory exhibited significant activation of the hypothalamus-pituitary-adrenal (HPA) axis (Buchanan et al., 2012; Engert et al., 2014). The SNS and HPA axis are two of the major physiological systems concurrently activated under conditions of acute stress. Activation of the SNS exerts a fast release of adrenaline and noradrenaline fostering a state of alertness and preparing the individual for a fight or flight response (Cannon, 1932). The slower response of the HPA axis stimulates the secretion of cortisol from the adrenal cortex, promoting the restoring of energy reserves (Selye, 1950). With regard to psychological indicators of empathic stress, research on emotional contagion (Elfenbein, 2014; Hatfield et al., 2014) provides numerous examples suggesting a transfer of affective states between individuals. However, studies on empathic stress have focused on the physiological stress response. So far, there is only one report of a combined assessment of physiological and self-report measures of acute stress (Erkens et al., 2019), showing increased subjectively perceived stress after observing a target in the Trier Social Stress Test (TSST).

Functionally, empathic stress, implying a transfer of stress-related information between individuals, enables them to understand the affective state of each other and to infer appropriate behavioral consequences (Engert et al., 2014). Waters et al. (2014) discussed multiple ways through which stress-related information could be transmitted in conditions of empathic stress. According to their suggestion, the observer receives cues from different sensory modalities, such as touch (Waters et al., 2017), visual, verbal or olfactory information, helping to get a reliable conception of the target’s state.

In this regard, the olfactory modality has been neglected in the field of empathic stress, even though it is increasingly attended with respect to its role in the transmission of stress-related information. Despite a long history of controversy (Wyatt, 2015), accumulating evidence supports the view that humans transfer social information *via* chemosensory communication (Lübke and Pause, 2015). It allows for an implicit way of human social communication that is considered relevant for reproduction and survival. The transmission of stress-related information, in particular, was termed one of the main functions of the olfactory system (Stevenson, 2010). There is meta-analytic evidence for chemosensory transfer of fear, stress and anxiety from one person to another (de Groot and Smeets, 2017). Of note in the context of empathic stress, an functional magnetic resonance imaging (fMRI) investigation found that chemosignals released in psychologically stressful situations differentially activate limbic brain regions that are critically involved in empathy-related processes (Prehn-Kristensen et al., 2009).

Research on chemosensory communication often examines complex mixtures of odor molecules (i.e., samples of axillary sweat), because the effect of a chemosignal does not solely depend on the constituting molecules, but also on their composition (Pause, 2012). However, to pinpoint the effects of a specific odorant *via* binding to specific receptor proteins, it can be useful to test chemical signals, which elicit a specific reaction (stereotyped behavior) that does not seem to require learning. Therefore, in our study, we pursued a monomolecular approach, investigating Hedione as a candidate chemosignal with respect to its effect on the empathic stress response. Hedione is a synthetically created aroma compound with a jasmine-like smell. Even though it is not released by humans, it seems to mimic the natural (unknown) molecules at the receptor site. Hedione is the first identified ligand of the human VN1R1 receptor (Wallrabenstein et al., 2015). This receptor is structurally homologous to a pheromone receptor in the rodent vomeronasal system (Boschat et al., 2002). Although there is a consensus that humans lack a functional vomeronasal organ (VNO; Smith et al., 2014), the VN1R1 receptor is one of five VNO receptor types that are still expressed in the human nasal mucosa (Rodriguez et al., 2000; Wallrabenstein et al., 2015). To what extent it is involved in human chemosignal detection is currently subject to investigation. Evidence from an fMRI study using Hedione to activate the VN1R1 receptor suggests stronger activations in limbic brain regions (amygdala, hippocampus) and the hypothalamus in contrast to a common floral odor (phenylethyl alcohol; Wallrabenstein et al., 2015). The hypothalamic activation, being ten times larger in females than in male participants, led the authors to consider a modulation of hormonal secretion by Hedione. A first behavioral study using Hedione showed increased positive and negative reciprocity in an economic game (Berger et al., 2017).

Given the special role of olfactory chemosignals in transmitting stress-related information, the hypothalamic activation by Hedione and the first evidence for its effect on reciprocal behavior, we hypothesized that Hedione would act in the sense of stress transmission by increasing the human empathic stress response on a physiological as well as a psychological level. For this purpose, we designed two experiments having participants observe another person in a stressful situation, either in a room scented with Hedione or in an odor-free control room. The ambient room odor was of similar valence in both conditions and participants were not aware that there was an odor distributed in the room, which makes this comparison provide an essential basis for the characterization of Hedione’s effect on empathic stress. To capture the stress response, we conducted repeated measurements of salivary cortisol, alpha-amylase and self-reported stress. Moreover, empathy with the targets was assessed on the dimensions of personal distress and empathic concern of the Emotional Response Scale (ERS).

## Materials and Methods

### Experiment 1

#### Participants

For an *a priori* sample size calculation with G-power 3.1.9.2 (Faul et al., 2007), parameter estimations were based on previous literature. We set out to determine an effect that is of the same size as the effect reported in the above-mentioned meta-analysis on human fear chemo-signaling (de Groot and Smeets, 2017; Hedges *g* = 0.36, corresponds to *f* = 0.18). Our estimate of the correlation between repeated measures was based on a previous study conducted in our laboratory using the TSST and the same assay to analyze the cortisol data (Langer et al., 2019). In this study, the correlation between repeated cortisol samples for the three time-points corresponding to our experiment resulted in *r* = 0.66. Accounting for a likely violation of the sphericity assumption, we chose a restrictive estimation of the nonsphericity correction coefficient (ε = 0.5). To detect the effect with an alpha error probability of 0.05 and a power of 80%, 60 participants were required.

Thus, 60 college students were tested of which 29 (*n* = 18 females) were randomly assigned to the control group and 31 (*n* = 17 females) to the Hedione group. In a telephone screening, we checked the eligibility of participants according to our predefined exclusion criteria. Participants should be aged 18–35 years with a Body Mass Index (BMI) from 16 to 30 kg/m^2^. None of them reported a history of psychological disorders, chronic or current illnesses, nor any current psychological or medical treatment. Further exclusion criteria were factors relevant for a functional sense of smell or the stress response, including a running nose, smoking, intake of medication and psychoactive drugs, breastfeeding, pregnancy, shift work, as well as vaccination, blood donation or traveling with time shift in the last month.

Participants were instructed to refrain from alcohol consumption and excessive physical activity 24 h before the experiment, from caffeinated drinks in the morning prior to the experiment, as well as from eating and drinking anything except for water 1 h prior to testing. Further, they were asked to resign the use of fragrant cosmetics on the testing day.

Amongst the female participants, 20 were free-cycling and 15 taking hormonal contraceptives. Further characteristics of the sample are depicted in Table 1. Participants were aged 19–35 years with BMI ranging from 16.4 to 30.5 kg/m^2^. There were no age- and BMI-related differences between the two groups, nor did they differ in self-reported trait empathy as measured by the interpersonal reactivity index (IRI). Perceived valence of the room odor did not differ between participants assigned to the Hedione and the control group. In the Hedione group, only three participants were aware of the odor dispersed in the room. In the control group, three participants had an incorrect guess, assuming they were assigned to the Hedione group.

**Table 1:** Sample characteristics displayed by the group.

	Control group		Hedione group		Group comparison		
	*M*	*SD*	*M*	*SD*	Test statistic	*p*	Effect size
Age in years	22.34	2.83	22.74	4.24	*W* = 472	0.74	*r* = 0.04
BMI in kg/m^2^	21.58	2.67	22.88	3.13	*t*_(58)_ = −1.72	0.09	*d* = 0.44
IRI	44.31	6.39	42.9	5.65	*W* = 545	0.16	*r* = 0.18
Odor valence	6.0	1.49	5.68	1.14	*W* = 498.5	0.43	*r* = 0.10

*Note: IRI, interpersonal reactivity index assessed with the Saarbrücker Persönlichkeitsfragebogen (Paulus, 2009). Valence (from 1 = unpleasant to 9 = pleasant) was rated on the Self-assessment Manikin (Bradley and Lang, 1994)*.

#### Experimental Procedure

**Figure 1:**
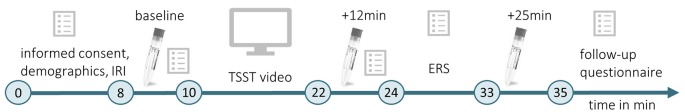
Timeline of Experiment 1. During the initial habituation phase (min 0–8), participants gave informed consent, completed a demographic questionnaire and the Interpersonal Reactivity Index (IRI; Paulus, 2009). The first baseline measurement of physiological and psychological stress indices was conducted (baseline, min 8–10) before participants watched a video of the Trier Social Stress Test (TSST; Kirschbaum et al., 1993, min 10–22). Subsequently, they provided another measurement of physiological and psychological stress indicators (min 22–24), which took place 12 min after the beginning of the video. After completing the Emotional Response Scale (ERS; Batson et al., 1997; min 24–33), the last physiological measurement was performed 25 min after the beginning of the TSST video (+25 min), and a follow-up questionnaire was completed.

Testings were conducted between 08:30 a.m. and 12:00 p.m. When entering the lab, a participant was placed in one of two identical chambers (1.70 × 2 × 3 m^3^). The chamber was either scented with Hedione (order# 947325, Firmenich, Meyrin, Switzerland, used as 5% solution in propylene glycol) by application of 5 ml on an Aroma Stream (AromaStream, TAOASIS GmbH, Detmold, Germany), or it was an odor-free control chamber, with 5 ml odorless propylene glycol applied to the Aroma Stream. The timeline of Experiment 1 is illustrated in Figure 1.

#### Induction of Empathic Stress

All participants watched the same, pre-recorded videotape showing a female target in the TSST (Kirschbaum et al., 1993). The TSST is a standardized laboratory protocol for the induction of psychosocial stress. It is composed of a 5-min preparation phase for a 5-min mock job interview followed by a 5-min mental arithmetic task. All of this is video-taped and takes place in front of a reserved committee consisting of a male and female member dressed in white lab coats. The committee is trained to act neutral, to follow standardized verbal instructions and to refrain from non-verbal feedback.

Participants were informed that the video would contain a recording of the TSST with a real participant in a job interview, who was observed by a trained committee. They were asked to attentively watch the video in order to rate their feelings and perception of the situation afterwards. The video was of 11.49-min duration starting with the TSST instruction followed by a 10 s blank screen signaling the start of the preparation phase. This phase was not part of the video. Instead, the TSST commenced directly after the blank screen with the 5-min mock job interview. During the subsequent mental arithmetic task, they counted backward from 2,043 in steps of 17. On the left half of the screen, a close-up of the target’s upper part of the body and on the right half, the committee was depicted. Each person seen in the video signed informed consent for the use of this video in the scope of the experiment.

#### Stress Measures

As a measure of subjectively perceived stress, a visual analog scale reaching from 0 (not stressed) to 100 (extremely stressed) was completed before and after the TSST video. To capture the physiological stress response, we collected salivary samples at three time points using Salivettes (Sarstedt, Nümbrecht, Germany). Out of these samples, concentrations of the hormone cortisol were extracted as a biomarker of HPA axis activity and the enzyme salivary alpha-amylase, which has been proposed to reflect SNS activity (Nater and Rohleder, 2009). In our choice of sampling time points (baseline, +12 min and +25 min), we considered that the SNS is rapidly activated due to stress, which leads to a fast increase of sAA levels. In our experiment, the response peak should be observed immediately after the TSST video at +12 min (Nater and Rohleder, 2009). This holds true for subjective stress, as well (Hellhammer and Schubert, 2012). Cortisol release, in turn, is slower and thus, we expect the highest cortisol levels at +25 min (Dickerson and Kemeny, 2004). Considering the data we actually obtained in both experiments, these time courses of the stress responses were supported (see “Manipulation Check” and “Empathic Stress” sections).

Salivary cortisol was analyzed on a Synergy2 plate reader (Biotek, USA) using commercial enzyme-linked immunosorbent assays (ELISAs; free cortisol in saliva; Demeditec, Germany) according to the manufacturer’s instructions. Intra- and inter-assay variability were less than 5% and 12%. A colorimetric test using 2-chloro-4-nitrophenyl-α-maltrotriosoide (CNP-G3) as a substrate reagent was applied to measure sAA concentration as described elsewhere (Lorentz et al., 1999). Intra- and inter-assay variabilities were below 6%.

#### Empathy Measures

Trait empathy was assessed in the initial habituation phase using the German Version of the IRI (Davis, 1980; Paulus, 2009). This questionnaire provides a multidimensional assessment of empathy on the dimensions fantasy, empathic concern, perspective taking and personal distress. Assuming state measures of empathy to be more sensitive to the experimental manipulation (see Engert et al., 2014), we used the ERS (Batson et al., 1997). It consists of 20 emotional adjectives capturing acute empathic concern and personal distress.

#### Confounding Variables

In a follow-up questionnaire, awareness of the experimental condition was tested by asking participants whether they thought to be in the control or Hedione group. Further, they rated the valence of the room odor on a 9-point pictorial rating scale ranging from unpleasant to pleasant.

#### Statistical Analysis

Statistical analyses were conducted using the software R (version 3.6.1) and RStudio (version 1.2.1335). At first, we calculated descriptive parameters of the sample and inspected boxplots for univariate outlier detection. Since no participant showed values higher than 3SD above the mean for the two VAS, all three cortisol or all three alpha-amylase measurements, we did not exclude any participant at this point to reduce the absolute size of bias caused by outlier removal (Miller, 1991). However, missing values for +12 min salivary cortisol and alpha-amylase led to exclusion of four participants from the respective analyses. Differences between Hedione and control group in terms of age, BMI, trait empathy (IRI) and odor valence ratings were checked *via* independent *t*-tests or, in case the assumptions were violated, non-parametric Mann–Whitney-*U*-tests (see Table 1).

Next, we tested our hypotheses assuming a stronger physiological and subjective stress response in the Hedione group as compared to the control group. For this purpose, a 2 × 2 ANOVA with the within-subjects factor Time (baseline, +12 min) and the between-subjects factor Group (Hedione/control) was conducted for the VAS, including all *N* = 60 participants. Likewise, 3 × 2 ANOVAs with the within-subjects factor Time (baseline, +12 min, +25 min) and the between-subjects factor Group (Hedione/control) were conducted for cortisol and alpha-amylase, including all *N* = 56 participants with a complete set of saliva samples. Since the assumption of normality was violated for all outcome variables, data were log-transformed. In case of a violated sphericity assumption, Greenhouse–Geisser correction was applied. To follow up on the main effect of time, pairwise *t*-tests were performed.

Moreover, we assessed differences between Hedione and control group in state empathic concern and personal distress, as obtained from the ERS using independent *t*-tests. Correlations between these two state empathy measures and the stress response were inspected using bivariate Pearson’s product-moment correlations. To capture the stress response, delta variables were created by subtracting baseline values from the peak values. For VAS (ΔVAS = VAS_+12 min_ − VAS_baseline_) and alpha-amylase (ΔAmylase = Amylase_+12 min_ − Amylase_baseline_), the peak was expected immediately after the TSST. For cortisol (ΔCort = Cort_+25 min_ − Cort_baseline_), we expected the peak to occur 25 min after onset of the stressor.

### Experiment 2

Three major changes were mades in Experiment 2. First, we aimed for a more homogeneous sample and tested only free-cycling women. This was due to a low response rate of women with oral contraceptive intake in Experiment 1 (see “Empathy” in women section), and a stronger hypothalamic activation by Hedione reported by Wallrabenstein et al. (2015). Second, participants did not watch the same pre-recorded video of a female participant in the TSST. Instead, they observed a male participant in the TSST *via* a live video. This enabled us to assess both components of empathic stress, vicarious stress, and stress resonance. For both, vicarious stress and stress resonance, we kept up our original hypotheses assuming an increase through Hedione. Third, we extended our measures of the subjective stress response assessing the VAS not only at baseline and +15 min, but also at +25 min. Further, the State Trait Anxiety Inventory (STAI-S; German version; (Laux et al., 1981) was added to measure changes in state anxiety evoked by the live TSST. As a secondary outcome measure, the Positive Affect Negative Affect Scale (PANAS; Watson et al., 1988) was used to characterize the time course of positive and negative affect throughout the experiment.

#### Participants

For Experiment 2, we performed a precise sample size calculation for the effects of interest based on the results obtained in Experiment 1. Since salivary cortisol was our main outcome, we inserted the effect size of the time × group interaction ($\eta_{\text{p}}^{2}$ = 0.023 corresponds to *f* = 0.153). The correlation among repeated measures among salivary cortisol was 0.89 in Experiment 1 and the nonsphericity correction coefficient ε = 0.63. For two groups and three-time points, a total sample size of 38 participants was found to be sufficient to detect the effect with an alpha error probability of 0.05 and a power of 95%.

We tested 37 female participants of which 18 were randomly assigned to the control group and 19 to the Hedione group. In addition, a sample of 16 male participants were recruited to act as subjects in the TSST. The sample again consisted of college students. We applied the same exclusion criteria as in Experiment 1, again checked in a standardized telephone screening prior to testing. Two participants deviated from our exclusion criteria (BMI > 30) due to the discrepancies of their data in the telephone screening and the demographic questionnaire. Since no conspicuous data points in the other variables were recorded for these participants, they were not excluded from the analyses.

Characteristics of the female observers are depicted in Table 2. Female observers were aged 19–28 years with BMI ranging from 18.34 to 32.87 kg/m^2^. There were no age- and BMI-related differences between the two groups and the distribution of menstrual cycle phases was approximately equal in the Hedione (five menstruation, four follicular, five ovulation, five luteal) and control group (two menstruation, five follicular, five ovulation, six luteal). Moreover, groups did neither differ in self-reported trait empathy as measured by the IRI nor in the perceived valence of the room odor. In the Hedione group, only two participants were aware of the odor dispersed in the room. In the control group, two participants had an incorrect guess, assuming they were assigned to the Hedione group.

**Table 2:** Sample characteristics of the female observers displayed by the group.

	Control group		Hedione group		Group comparison		
	*M*	*SD*	*M*	*SD*	Test statistic	*p*-value	Effect size
Age in years	20.78	1.66	21.37	2.65	*W* =161	0.77	*r* = −0.05
BMI in kg/m^2^	23.65	3.94	21.79	2.23	*t*_(35)_ = 1.78	0.08	*d* = −0.59
IRI	44.72	4.76	45.79	4.22	*t*_(35)_ = −0.72	0.48	*d* = 0.24
Odor valence	6.5	1.25	6.16	1.64	*W* = 213	0.19	*r* = 0.22

*Note: IRI, interpersonal reactivity index assessed with the Saarbrücker Persönlichkeitsfragebogen (Paulus, 2009). Valence (from 1 = unpleasant to 9 = pleasant) was rated on the Self-Assessment Manikin (Bradley and Lang, 1994)*.

Male targets were aged between 19 and 26 years (*M* = 21.8, *SD* = 2.31) with BMI ranging from 19.79 to 25.99 kg/m^2^ (*M* = 23.75, *SD* = 1.74).

#### Experimental Design and Procedure

**Figure 2:**
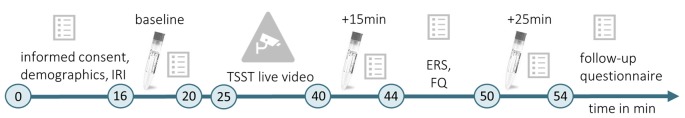
Timeline of Experiment 2. During the initial habituation phase (min 0–16), participants gave informed consent, completed a demographic questionnaire and the IRI (German version; Paulus, 2009). Then, the first baseline measurement of the physiological and psychological stress indices was conducted (baseline, min 16–20). The committee for the TSST (Kirschbaum et al., 1993) entered the testing room where the male target was seated, the live video was started and TSST instructions were delivered (min 20–25). The TSST (min 25–40) was followed by a second measurement of physiological and psychological stress indicators (min 40–44), which took place 15 min after the beginning of the video. Subsequently, the ERS (Batson et al., 1997) and a self-developed familiarity questionnaire (FQ) were completed (min 44–50). The last stress measurement (min 50–54) took place 25 min after the beginning of the TSST live video (+25 min). For male targets and female observers, the experiment followed the same timeline. However, male targets did not complete the IRI, ERS, and FQ. In the end, female observers completed a follow-up questionnaire.

Testings were conducted between 9:00 a.m. and 12:00 p.m. This time, up to three female participants could be tested simultaneously. When entering the lab, the first experimenter placed them in one of three identical chambers (1.70 × 2 × 3 m^3^). The chamber was either scented with Hedione (order# 947325, Firmenich, Meyrin, Switzerland, used as 5% solution in propylene glycol) by application of 5 ml on an Aroma Stream (AromaStream, TAOASIS GmbH, Detmold, Germany), or it was an odor-free control chamber, with 5 ml odorless propylene glycol applied to the Aroma Stream. At the same time, the second experimenter supervised a male participant in a testing room next door. In this room, an IP Camera (EDIMAX IC-3140W, Conrad Electronic SE, Hirschau, Germany) was installed, allowing for simultaneous video and sound broadcasting to the three experimental chambers. The timeline of Experiment 2 is illustrated in Figure 2.

#### Induction of Empathic Stress

Instead of the pre-recorded videotape used in Experiment 1, female observers watched a live video of a male participant undergoing the TSST. It was broadcasted *via* an IP camera placed in the right upper corner of the TSST room, capturing the back of the two committee members, as well as the upper part of the male target’s body. After the start of the live video, the second experimenter delivered the TSST instructions to the male target and left the room. Subsequently, the TSST started with 5-min preparation time, a 5-min mock job interview and a 5-min mental arithmetic task. After the TSST, the second experimenter re-entered the TSST room, while the committee left and the live video was stopped. Other than the video used in Experiment 1, participants also observed the preparation time, since it was not possible to interrupt the broadcasting. Most importantly, the active elements of the TSST (i.e., free speech and mental arithmetic task) had exactly the same length. In order to maximize the comparability of the situations observed by the female participants, the TSSTs were highly standardized. In each TSST, the same committee members were present. They were trained to use standardized verbal responses and to act in a neutral and reserved way. Moreover, the same standardized instructions were read out by the experimenter before the TSST and the experimental setting was identical (room, camera position, timing).

#### Stress Measures

For a more elaborate investigation of the psychological stress response in this experiment, the VAS was completed at all three time points. Further, the state scale of the STAI-S was added. It assesses state anxiety by posing 20 statements (i.e., “I feel nervous”) for which participants rate their level of agreement on a four-point Likert scale. To assess positive and negative affect with ten items each on a five-point Likert scale, the PANAS was completed three times by each participant.

As in Experiment 1, physiological reactivity to the TSST observation was assessed *via* salivary samples collected at three different time points to extract cortisol and alpha-amylase. To determine cortisol concentrations in the saliva samples, a time-resolved fluorescence immunoassay was used. The intra-assay coefficient of variation was below 4.0%, and the corresponding inter-assay coefficients of variation were below 12.2%. A colorimetric test using 2-chloro-4-nitrophenyl-α-maltrotriosoide (CNP-G3) as a substrate reagent was applied to measure sAA concentration as described elsewhere (Lorentz et al., 1999). Intra- and inter-assay variability was below 9%.

#### Empathy Measures

We applied the same trait (Paulus, 2009) and state (Batson et al., 1997) empathy questionnaires as in Experiment 1.

#### Confounding Variables

Again, a follow-up questionnaire was completed at the end of the experiment, assessing awareness of the experimental condition and valence of the room odor. Moreover, participants rated familiarity with the targets using a self-developed familiarity questionnaire (FQ). The first two questions were dichotomous yes-or-no questions as to whether the target was familiar or related. If familiar with the target, observers were asked to state how often they meet on a weekly basis (0, 1–2, 3–4, >4) and for which purpose (private or occupational). They also rated closeness to the target (from not close to very close) and how they rate the target (from very negative to very positive) on 7-point Likert Scales.

#### Statistical Analysis

At first, we calculated descriptive parameters of the sample and inspected boxplots for univariate outlier detection. One participant showing extremely high alpha-amylase values (>3 SD above the mean) at all time points was excluded from analyses containing alpha-amylase as an outcome measure. Differences between Hedione and control group in terms of age, BMI, trait empathy (IRI) and odor valence ratings were checked using independent *t*-tests or, in case the assumptions were violated, the non-parametric Mann–Whitney-*U*-test (see Table 2).

To check whether stress induction was successful, repeated measures ANOVAs with the within-subjects factor Time (baseline, +12 min, +25 min) were conducted for cortisol, alpha-amylase and subjective stress measured in the male sample. Due to missing data of one participant, the analyses were conducted with *N* = 15 participants. The main effects of time were followed up using pairwise comparisons. Since VAS data were not normally distributed, they were log-transformed.

Next, we inspected the observer’s stress responses. For this purpose, 3 × 2 ANOVAs with the within-subjects factor Time (baseline, +12 min, +25 min) and the between-subjects factor Group (Hedione/control) were conducted for VAS, STAI-S, PANAS positive affect scale and cortisol including all *N* = 37 participants and for alpha-amylase with *N* = 36 participants. The assumption of normality was violated for cortisol and VAS data, which were therefore log-transformed. In case of a violated sphericity assumption, Greenhouse–Geisser correction was applied. To follow up on the main effect of time, pairwise *t*-tests were performed. To follow up a Time × Group interaction, repeated measures ANOVAs with Time as a within-subjects factor were separately calculated for the two groups. For the PANAS negative affect scale, we report Bonferroni-corrected non-parametric Mann–Whitney-*U-tests* comparing the two groups at all three sampling points. This was required due to violation of the normality assumption and the assumption of homogeneity of the variance-covariance matrices, and due to a lack of non-parametric alternatives to a repeated-measures ANOVA with within and between factors.

In some cases, two participants were tested subsequently in the same experimental chamber (with a delay of 2 h between the onset of the sessions), meaning that the second participant could have been exposed to a higher concentration of Hedione. To rule out a potential accumulative effect of exposure to Hedione, session order was added as a covariate in case an effect of Hedione was detected in the 3 × 2 ANOVAs. In the same way, we checked for an effect of awareness of the experimental condition.

Other than in Experiment 1, we were able to assess the effect of Hedione on both, vicarious stress and stress resonance. For this purpose, the statistical analyses used by Engert et al. (2014) were adapted to assess the empathic stress response. This included calculation of change scores for cortisol (ΔCort = Cort_+25 min_ − Cort_baseline_), alpha-amylase (ΔAmylase = Amylase_+15 min_ − Amylase_baseline_), VAS (ΔVAS = VAS_+15 min_ − VAS_baseline_) and STAI (ΔSTAI = STAI_+15 min_ − STAI_baseline_). As in Experiment 1, delta scores were calculated by subtracting baseline values from the peak values. These delta scores were calculated for both, targets and observers and corrected for baseline levels (see Engert et al., 2014). Subsequently, linear regression models using restricted maximum likelihood (REML) estimates were tested. These models included Group, targets’ delta scores, as well as their interaction as predictors for observers’ delta scores. In these models, modulation of vicarious stress by Hedione would become manifest as an effect of group. An effect of the targets’ delta scores or interaction of Group and targets’ delta scores would indicate a stress resonance between target and observer. Model fit was quantified by the likelihood-based coefficient R^2^. Due to missing data of one male target, *N* = 34 female observers were included in the analyses.

As in Experiment 1, differences between Hedione and control group in state empathic concern and personal distress were assessed using independent *t*-tests. Correlations between these two state empathy measures and the observers’ delta scores were inspected using bivariate Pearson’s product-moment correlations.

Additionally, to check for possible mechanisms associated with the effects of Hedione, we conducted explorative analyses assessing bivariate Pearson’s product-moment correlations between odor valence and observers’ VAS and STAI-S delta scores, as well as cortisol response and observers’ VAS and STAI-S delta scores.

## Results

### Experiment 1

#### Empathic Stress

**Figure 3:**
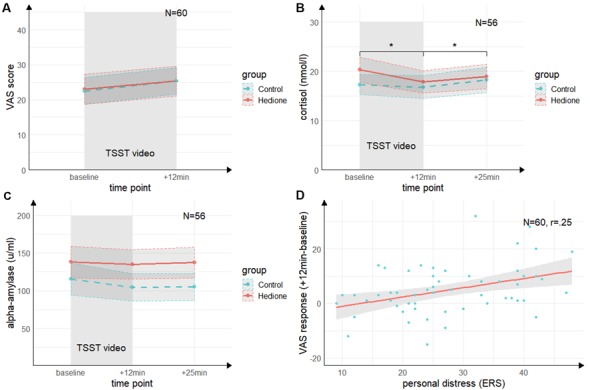
Time course of stress levels in Experiment 1. **(A)** Ratings of the visual analog scale (VAS) did not change from baseline to directly after the TSST (+12 min). There were no differences between the stress and control groups. **(B)** Salivary cortisol levels decreased from baseline (baseline) to directly after the TSST (+12 min) and increased again 25 min after (+25 min) the beginning of TSST. There were no differences between the stress and control groups. **(C)** Salivary alpha-amylase levels did not change from baseline (baseline) to directly after the TSST (+12 min) and 25 min after (+25 min) the beginning of TSST. There were no differences between the stress and control groups. **(D)** There was a trend towards a positive correlation of the subscale personal distress of the ERS with the VAS response. *Note*: *p < 0.05. Shaded errors denote *SEM*.

There was no main effect of Time (*F*_(1,58)_ = 2.69, *p* = 0.11, $\eta_{\text{p}}^{2}$ = 04), no main effect of Group (*F*_(1,58)_ = 0.19, *p* = 0.66, $\eta_{\text{p}}^{2}$ = 0.00), and no Time × Group interaction (*F*_(1,58)_ = 0.10, *p* = 0.76, $\eta_{\text{p}}^{2}$ = 0.00) for the VAS revealed by the 2 (Time) × 2 (Group) ANOVA (see Figure 3A).

The 3 (Time) × 2 (Group) ANOVA for salivary cortisol (see Figure 3B) revealed a trend towards a main effect of Time (*F*_(1.26,67.99)_ = 2.22, *p* = 0.06, $\eta_{\text{p}}^{2}$ = 0.06), which was driven by lower cortisol levels at +12 min as compared to baseline (*t*_(54)_ = 2.88, *p* = 0.01, *d* = 0.39) and +25 min (*t*_(54)_ = 2.99, *p* = 0.01, *d* = 0.40). There was neither a significant main effect of Group (*F*_(1,54)_ = 0.12, *p* = 0.73, $\eta_{\text{p}}^{2}$ = 0.00), nor a Time × Group interaction (*F*_(1.26,67.99)_ = 1.27, *p* = 0.27, $\eta_{\text{p}}^{2}$ = 0.02).

For alpha-amylase, the 3 (Time) × 2 (Group) ANOVA (see Figure 3C) yielded neither significant main effects of Time (*F*_(2,108)_ = 0.22, *p* = 0.80, $\eta_{\text{p}}^{2}$ = 0.00) and Group (*F*_(1,54)_ = 0.55, *p* = 0.46, $\eta_{\text{p}}^{2}$ = 0.01), nor an interaction of these factors (*F*_(2,108)_ = 0.84, *p* = 0.43, $\eta_{\text{p}}^{2}$ = 0.02).

To further elucidate our null findings for the effects of interest (i.e., the Time × Group interactions), we additionally applied Bayesian hypothesis testing to quantify the relative evidence for the null hypothesis using JASP 0.11.1.0. We conducted Bayesian repeated-measures ANOVAs comparing the effects of the Time × Group interaction against the null model using a prior for fixed effects with r scale prior width of 0.5. The Bayes factors provide moderate evidence favoring the null hypothesis for cortisol (*BF*_(01)_ = 4.70), strong evidence for the VAS (*BF*_(01)_ = 16.00) and very strong for alpha-amylase (*BF*_(01)_ = 85.93).

#### Empathy

Both groups did neither differ in state empathic concern (*t*_(58)_ = −0.85, *p* = 0.40, *d* = 0.22), nor in-state personal distress (*t*_(58)_ = −0.22, *p* = 0.83, *d* = 0.06) as measured by the ERS. Moreover, there were no significant correlations between cortisol or alpha-amylase and the ERS indices (*p*’s > 0.05). For the VAS response, there was a trend towards a positive correlation with personal distress (*r* = 0.25, *t*_(58)_ = 1.95, *p* = 0.06; see Figure 3D, but not with empathic concern (*r* = 0.15, *t*_(58)_ = 1.13, *p* = 0.26). This indicates a non-significant trend towards an association of higher subjective stress ratings with a higher degree of direct personal distress felt due to the observation, but not with higher empathic concern for the target.

### Experiment 2

#### Manipulation Check

For the male targets, repeated measures ANOVAs with Time as within-subjects factor revealed main effects of Time for VAS (*F*_(2,28)_ = 20.61, *p* < 0.001, $\eta_{\text{p}}^{2}$ = 0.60), STAI-S (*F*_(2,28)_ = 10.97, *p* < 0.001, $\eta_{\text{p}}^{2}$ = 0.44), cortisol (*F*_(2,28)_ = 19.40, *p* < 0.001, $\eta_{\text{p}}^{2}$ = 0.58) and alpha-amylase (*F*_(2,28)_ = 22.26, *p* < 0.001, $\eta_{\text{p}}^{2}$ = 0.61). For salivary cortisol, pairwise *t*-tests revealed that it was driven by higher cortisol levels at +25 min, as compared to baseline (*t*_(13)_ = 4.95, *p* < 0.001, *d* = 1.28) and +15 min (*t*_(13)_ = −5.28, *p* < 0.001, *d* = −1.36). For VAS (*t*_(13)_ = 5.85, *p* < 0.001, *d* = 1.51), STAI-S (*t*_(13)_ = 3.59, *p* = 0.01, *d* = 0.93) and salivary alpha-amylase (*t*_(13)_ = 6.66, *p* < 0.001, *d* = 1.72), +15 min scores were higher than baseline, as well as than +25 min scores for VAS (*t*_(13)_ = 5.41, *p* < 0.001, *d* = 1.40), STAI-S (*t*_(13)_ = 4.72, *p* < 0.001, *d* = 1.22) and salivary alpha-amylase (*t*_(13)_ = 4.7, *p* < 0.001, *d* = 1.22). Based on these results, we conclude that the TSST effectively evoked a physiological and psychological stress response in the male targets.

#### Empathic Stress

**Figure 4:**
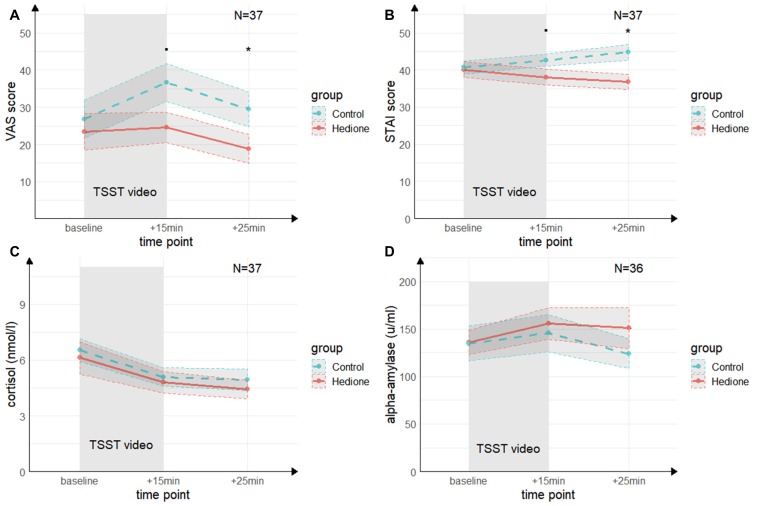
Group differences in empathic stress in Experiment 2. **(A)** Ratings of the visual analog scale (VAS) tend to be higher in the control group directly after the TSST (+15 min) and are significantly higher in the control group 25 min after (+25 min) the beginning of TSST. **(B)** Ratings of the state scale of the State Trait Anxiety Inventory (STAI) tend to be higher in the control group directly after the TSST (+15 min) and are significantly higher in the control group 25 min after (+25 min) the beginning of TSST. **(C)** There were no group differences in salivary cortisol levels and **(D)** in salivary alpha-amylase levels at neither time point. *Note*: ^•^*p* = 0.06, *p < 0.05. Shaded errors denote *SEM*.

A main effect of Time (*F*_(2,70)_ = 4.68, *p* = < 0.01, $\eta_{\text{p}}^{2}$ = 0.12) for the VAS was revealed by the 3 (Time) × 2 (Group) ANOVA (see Figure 4A). It was driven by higher VAS scores at +15 min compared to +25 min (*t*_(35)_ = 3.24, *p* < 0.01, *d* = 0.53) and, on a trend-level compared to baseline (*t*_(35)_ = 2.46, *p* = 0.06, *d* = 0.40). There was a trend towards a main effect of Group (*F*_(1,35)_ = 2.96, *p* = 0.09, $\eta_{\text{p}}^{2}$ = 0.08), which remained relatively stable when adding session order (*F*_(1,35)_ = 2.83, *p* = 0.10, $\eta_{\text{p}}^{2}$ = 0.07) or awareness (*F*_(1,35)_ = 2.83, *p* = 0.10, $\eta_{\text{p}}^{2}$ = 0.07) as a covariate. VAS scores at +15 min tended to be lower in the Hedione group (*M* = 24.63, *SD* = 17.99) than in the control group (*M* = 36.67, *SD* = 21.91, *t*_(35)_ = 1.96, *p* = 0.06) and were significantly lower at +25 min in the Hedione group (*M* = 18.79, *SD* = 17.15) as compared to the control group (*M* = 29.5, *SD* = 19.78, *t*_(35)_ = 2.12, *p* = 0.04). No Time × Group interaction (*F*_(2,70)_ = 1.79, *p* = 0.17, $\eta_{\text{p}}^{2}$ = 0.05) was found.For the STAI, the 3 (Time) × 2 (Group) ANOVA (see Figure 4B) resulted in a Time × Group interaction (*F*_(1.39,48.73)_ = 3.24, *p* = 0.02, $\eta_{\text{p}}^{2}$ = 0.12). This effect remained stable when adding session order (*F*_(1.39,48.73)_ = 3.24, *p* = 0.02, $\eta_{\text{p}}^{2}$ = 0.12) or awareness (*F*_(1.39,48.73)_ = 3.24, *p* = 0.02, $\eta_{\text{p}}^{2}$= 0.12) as a covariate. Conducting one-way ANOVAs with the within-subjects factor time separately for the two groups, it turned out that a main effect of time was only found in the Hedione group (*F*_(2,36)_ = 3.89, *p* = 0.03, $\eta_{\text{p}}^{2}$ = 0.18). It was driven by lower scores at +25 min as compared to baseline (*t*_(16)_ = −2.67, *p* < 0.05, *d* = −0.61). STAI scores at +15 min differed only on a trend level between groups (*t*_(35)_ = 1.74, *p* = 0.09, *d* = −0.57). At +25 min, they were significantly lower in the Hedione group (*M* = 36.79, *SD* = 8.63) than in the control group than in the control group (*M* = 44.78, *SD* = 9.12, *t*_(35)_ = 2.74, *p* < 0.01, *d* = −0.90). No main effect of Time (*F*_(1.39,48.73)_ = 0.05, *p* = 0.86, $\eta_{\text{p}}^{2}$ = 0.00) or Group (*F*_(1,35)_ = 3.27, *p* = 0.08, $\eta_{\text{p}}^{2}$ = 0.09) yielded significance.

Mann–Whitney-*U*-Tests comparing the values obtained in the PANAS negative effect subscale between the two groups at each sampling point showed no differences between the groups at baseline (*W* = 193.5, *p* = 0.50, *r* = 0.11) and +15 min (*W* = 218.5, *p* = 0.15, *r* = 0.24), and a trend-level difference at +25 min (*W* = 226.5, *p* = 0.09, *r* = 28). For positive affect, no main effect of Time (*F*_(1.59,55.69)_ = 0.97, *p* = 0.30, $\eta_{\text{p}}^{2}$ = 0.03), no Group effect (*F*_(1,35)_ = 1.10, *p* = 0.30, $\eta_{\text{p}}^{2}$ = 0.03) and no Time × Group interaction (*F*_(1.59,55.69)_ = 0.52, *p* = 0.9, $\eta_{\text{p}}^{2}$ = 0.02) were found.

The 3 (Time) × 2 (Group) ANOVA for salivary cortisol (see Figure 4C) revealed a main effect of Time (*F*_(1.60,56.08)_ = 27.96, *p* < 0.001, $\eta_{\text{p}}^{2}$ = 0.50), which was driven by a decrease in cortisol levels from baseline to +15 min (*t*_(35)_ = −6.73, *p* < 0.001, *d* = −1.11) and +25 min (*t*_(35)_ = −6.59, *p* < 0.001, *d* = −1.08). There was neither a significant main effect of Group (*F*_(1,35)_ = 0.53, *p* = 0.0.47, $\eta_{\text{p}}^{2}$ = 0.02), nor a Time × Group interaction (*F*_(1.60,56.08)_ = 0.20, *p* = 0.73, $\eta_{\text{p}}^{2}$ = 0.01).

For alpha-Amylase (see Figure 4D), there were no effects of Time (*F*_(1.54,52.35)_ = 2.11, *p* = 0.09, $\eta_{\text{p}}^{2}$ = 0.07), Group (*F*_(1,34)_ = 0.29, *p* = 0.59, $\eta_{\text{p}}^{2}$ = 0.01), and no interaction of these factors (*F*_(1.54,52.35)_ = 1.30, *p* = 0.20, $\eta_{\text{p}}^{2}$ = 0.05).

Results of the linear regression models including Group, target’s delta scores, as well as their interaction as predictors for observer’s delta scores are displayed in Table 3 (*N* = 34). For salivary cortisol and salivary alpha-amylase, none of the three predictors explained substantial variance in the observer’s delta scores. For the subjective measures (VAS and STAI-S), observer’s delta scores were predicted by Group, indicating higher subjective vicarious stress in the control than in the Hedione group.

**Table 3:** Parameter estimates and fit statistics for cortisol, alpha-amylase, VAS and STAI-S models.

	Cortisol	Alpha-Amylase	VAS	STAI-S
Intercept	−0.165	18.617	16.810	2.955
Group	0.168	−17.776	−12.798*	−4.664^•^
ΔTarget	0.102	−0.083	−0.315	−0.261
Group × ΔTarget	0.031	0.297	0.125	0.165
Model Fit: R^2^	0.136	0.092	0.170	0.156

Note: ΔTarget, corrected difference scores from baseline to peak of the target’s response. ^•^*p* = 0.05, *p < 0.05.

#### Empathy

**Figure 5:**
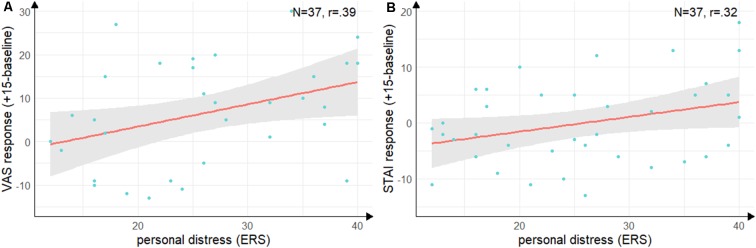
Correlations between personal distress and subjective stress response. **(A)** Correlation of the subscale personal distress of the ERS with difference scores on the visual analog scale from baseline (baseline) to peak (+15 min). **(B)** Correlation of the subscale personal distress with the difference scores on the state scale of the STAI from baseline (baseline) to directly after the TSST (+15 min). *Note*: shaded errors denote *SEM*.

Groups did not differ in state empathic concern (*t*_(35)_ = −1.09, *p* = 0.28, *d* = −0.35), but with a large effect size in-state personal distress (*t*_(35)_ = −2.55, *p* = 0.02, *d* = −0.84) with lower personal distress in the Hedione group. There were no significant correlations between the cortisol, alpha-Amylase the ERS indices. For the VAS response (see Figure 5A), there was a positive correlation with personal distress (*r* = 0.39, *t*_(35)_ = 2.52, *p* = 0.02) and for the STAI response (see Figure 5B), there was a trend into the same direction (*r* = 0.32, *t*_(35)_ = 2.00, *p* = 0.05). Thus, in accordance with Experiment 1, higher subjective stress ratings were associated with a higher degree of direct personal distress felt due to the observation, but not with higher empathic concern for the target.

#### Explorative Analyses

Observer’s VAS and STAI-S responses were neither correlated with odor valence, nor with cortisol responses (*p*’s > 0.05).

## Discussion

In the present study, we investigated the effects of the odorant Hedione on physiological and psychological empathic stress. For this purpose, two experiments were designed, each testing one group of participants in an odor-free room and a second group in a room scented with Hedione. In Experiment 1, participants watched a video of a female target in the TSST. In Experiment 2, they watched a live video of a male target in the TSST. Our results show a reduction of subjective vicarious stress by Hedione. The subjective stress response evoked by observing the TSST was associated with personal distress, but not with empathic concern. Observers showed only weak or no physiological empathic stress responses, which were unaffected by Hedione.

Hedione did not increase the empathic stress response in the sense of transmission of stress-related information. Instead, it reduced subjective vicarious stress and state anxiety in response to observing a stressful situation. This indicates a stress-buffering effect of Hedione as it has been reported for odorants that are used in aromatherapy (Herz, 2009; Hur et al., 2014). The effects of aromatherapy are best accounted for by a psychological explanation. It claims that the individual response to an odor depends on its learned associative value, with perceived odor quality being the most relevant factor for determining the emotional and physiological response (Herz, 2009). Contrary to this, we found changes in subjective stress to be unrelated to odor valence. The effect of Hedione may therefore not be of a mere psychological nature. Previous research led to speculations about possible hormonal effects as a result of hypothalamic activation by Hedione (Wallrabenstein et al., 2015). However, given the correlative results presented above, the effect of Hedione on the subjective vicarious stress response is probably not modulated by the stress hormone cortisol. To uncover the psychological or physiological processes behind the observed effect, future studies should explore other hormonal mechanisms, such as the release of oxytocin. This peptide hormone was repeatedly shown to be involved in social behavior (Heinrichs et al., 2009) and was found to improve the ability to infer the mental state of another person from viewing a picture of their eyes (Domes et al., 2007). On the other hand, it reduced the behavioral and neural response to chemosensory stress cues by downregulating stress-induced activations of the limbic system while strengthening top-down control (Maier et al., 2018). Oxytocin is thus an interesting candidate hormone that might be involved in the stress-alleviating effect of Hedione on empathic stress.

Our findings could suggest that a response to the stressor is required for Hedione to exert its effects. The reduction of subjective vicarious stress was only found in Experiment 2, where observers showed an increase in subjective stress due to the video. In Experiment 1, there was neither an increase in subjectively perceived stress, nor any effect of Hedione. The same holds true for physiological responses in the observers, which were either weak or absent and not affected by Hedione. In order to complement our reasoning, we retrospectively inspected Bayes factors, which quantify the relative evidence for the null and alternative hypothesis. In line with our explanation, the respective Bayes factors for our effects of interest speak in favor of the null hypothesis, suggesting moderate to very strong evidence. We propose that this absence of Hedione effects could be explained by the absence of a stress response in the first place. Likewise, no stress resonance became evident in Experiment 2, and no modulation of stress resonance by Hedione. Most likely, this was due to the combination of unfamiliarity with the targets and a virtual broadcasting of the stressful situation instead of a real-life observation (Engert et al., 2014). We, therefore, propose that aspects of the empathic stress response need to be sufficiently pronounced for Hedione to affect them. A next step towards the characterization of Hedione’s effects on the empathic stress response could be to intensify the observational setting, for instance by observing emotionally close others instead of strangers and by establishing a real-life observation. In this regard, assessing physiological and psychological stress prior to the experiment would help to assure that no anticipatory arousal differences between the groups due to unsuccessful randomization exist, which may mask an effect of Hedione on the stress response.

To unravel the adaptive function of Hedione reducing subjective vicarious stress, two considerations arise from our investigation. First, the reduction of subjective vicarious stress started to emerge directly after observation of the target being stressed, and it became even more pronounced over time. This argues for a rather persistent effect of Hedione that continues beyond the critical situation itself. Possibly, it could be relevant for later processing and behavioral consequences of the situation. Second, the close associations between subjective stress measures and personal distress could be of interest. Personal distress refers to the observer’s direct feelings of distress. It represents a self-oriented aversive emotional response, presumably arising from imagining the self in a situation of distress (Batson et al., 1997). By reducing personal distress, exposure to Hedione prevented the observers from strongly immersing in their own emotional response while observing another person being stressed. Meanwhile, empathic concern, denoting the extent to which observers felt with the target, was unaffected by Hedione, and not associated with subjectively perceived stress. Based on these findings we suggest that the reduction of subjective vicarious stress by Hedione is a manifestation of reduced personal distress of the observers, and not related to their empathic concern.

In this respect, it would further be interesting to examine related behavioral and motivational aspects. Personal distress was found to evoke egoistic motivation (Batson et al., 1987) and to negatively affect prosocial behavior (Eisenberg et al., 1989). This raises the question of whether exposure to Hedione would go along with reduced egoistic motivation and more prosocial behavior. Interestingly, stress itself was also found to affect both, egoistic motivation (Tomova et al., 2014) and prosocial behavior (von Dawans et al., 2012, 2019) in a sex-dependent manner. At the same time, stress increased emotional empathy in healthy men (Wolf et al., 2015) and women (Wingenfeld et al., 2018). Future research on empathic stress should, therefore, address motivational and behavioral consequences with respect to the interactive effects of Hedione and stress, considering possible sex effects.

Assessing potential sex differences is of similar relevance from a chemosensory point of view, as Hedione activated the hypothalamus more strongly in female participants (Wallrabenstein et al., 2015). Moreover, some effects of chemosensory stress signals appear to be sex-specific, such as the reduction of positive emotional priming by chemosensory anxiety cues, which was only found in women (Pause et al., 2004). In the context of the current study, a sex-dependency of the effect is conceivable since it was observed in a female sample only and can thus not be extrapolated to men. A potential sex difference should, therefore, be specified in future studies. Likewise, the menstrual cycle phase constitutes an important modulator of the stress response (Kudielka and Kirschbaum, 2005; Merz and Wolf, 2017) and chemosensory effects. It was, for instance, demonstrated that free-cycling women differ in sensitivity to specific odors depending on the menstrual cycle phase (Lundström et al., 2006). In the fertile phase, they were more sensitive to a social odorant (androstadienone) than in the nonfertile phase, whereas sensitivity for an environmental odor (PEA) did not differ. Considering these findings and further complex interactions between reproductive hormones and olfactory function (for a review, see Doty and Cameron, 2009), the menstrual cycle phase is an important factor to be considered in further characterization of the effect reported here.

One methodological aspect worth discussing is that testing was conducted in the morning. Especially in Experiment 2, we observe a decreasing pattern in cortisol over time, resembling the circadian cortisol rhythm. Due to practical reasons, testing in the afternoon was not possible in this study but is recommended for potentially more sensitive measurement of cortisol responses to empathic stress in future research (Dickerson and Kemeny, 2004). Further, we did not control for possible sex differences, menstrual cycle phase or oral contraception in Experiment 1 (Kudielka and Kirschbaum, 2005), whereas we limited our sample to free-cycling female observers and male targets in Experiment 2. Even though observer sex did not modulate empathic stress (Engert et al., 2014), a systematic variation of opposite and same-sex dyads is missing in the field of empathic stress and might produce differential effects, which are not represented by the current study. Following the example of the two studies testing effects of Hedione in humans (Wallrabenstein et al., 2015; Berger et al., 2017), we did not screen participants for specific anosmia (Croy et al., 2015) to Hedione. However, this represents a limitation to attributing the effects specific to the odorant itself. Moreover, even though Hedione is a likely candidate chemosignal, it does not occur in a human body fluid. To extend our knowledge about the transfer of stress-related information under conditions of empathic stress, studies are needed that consider other chemosignals, i.e., social body odors released in stressful conditions.

To sum up, the present study shows that Hedione reduces subjective vicarious stress. As stated in the “Discussion” section, it remains to be clarified, whether the odorant could also affect physiological vicarious stress and stress resonance. Based on the findings presented above, we conclude that Hedione, by reducing personal distress, prevented a strong emotional response of the observers while leaving empathic concern with the target unaffected. Future research is warranted to clarify functional and motivational aspects, as well as the mechanisms underlying this effect.

## Data Availability Statement

The datasets of experiments 1 and 2, as well as the R code for statistical analyses and figures, can be found in the OSF (https://osf.io/pyjg7/?view_only=da0a387cfaf84afc8484d0154c936812).

## Ethics Statement

This study was carried out in accordance with the recommendations of the German Psychological Society (DGPs). The protocol was approved by the local ethical committee of the Faculty of Psychology at the Ruhr University Bochum (Proposal No. 434). All participants gave written informed consent and all procedures were in accordance with the Declaration of Helsinki.

## Author Contributions

AP and OW contributed to the conception and design of the study. AP was responsible for data acquisition, performed the statistical analysis and drafted the article. OW, MB and HH revised the manuscript. All authors read and approved the submitted version and are accountable for the content of the work.

## Conflict of Interest

The authors declare that the research was conducted in the absence of any commercial or financial relationships that could be construed as a potential conflict of interest.
